# Antihyperglycemic and Antihyperlipidemic Activity of Hydroponic* Stevia rebaudiana* Aqueous Extract in Hyperglycemia Induced by Immobilization Stress in Rabbits

**DOI:** 10.1155/2017/9251358

**Published:** 2017-07-03

**Authors:** Anush Aghajanyan, Zaruhi Movsisyan, Armen Trchounian

**Affiliations:** ^1^Department of Biochemistry, Microbiology and Biotechnology, Faculty of Biology, Yerevan State University, 0025 Yerevan, Armenia; ^2^Research Institute of Biology, Faculty of Biology, Yerevan State University, 0025 Yerevan, Armenia

## Abstract

Diabetes mellitus (DM) is a serious worldwide problem related to human hyperglycemia. Thus, herbal preparations with antihyperglycemic properties especially leaf extracts of hydroponic* Stevia rebaudiana *(SR) would be useful in hyperglycemia treatment. The antihyperglycemic potential of this medicinal plant grown using hydroponics methods has been evaluated. Significant reduction of some biochemical characteristics for sugars and fatty acids in blood, liver, and muscle especially fasting glucose levels, serum triglycerides, LDL-cholesterol, total cholesterol levels, and increased HDL-cholesterol ones was shown with SR aqueous extract treatment. Therefore, the aqueous extract of SR is suggested to have antihyperglycemic and antihyperlipidemic activity and to restore liver and muscle glycogen levels (hepatoprotective effects) in hyperglycemia induced by immobilization stress in rabbits and might be recommended for treatment of DM (hyperglycemia).

## 1. Introduction

Diabetes mellitus (DM) is a global problem of the people over the world. The World Health Organization estimates that diabetes affects 285 mln people globally, and current predictions estimate that 438 mln people will be diabetic by 2030 [1]. DM is a metabolic disorder of the endocrine system, and it is characterized by hyperglycemia resulting in an increased hepatic glucose production, diminished insulin secretion, and impaired insulin action. It has adverse effect on carbohydrate, lipid, and protein metabolism resulting in chronic hyperglycemia and abnormality of lipid profile. These lead to series of secondary complications including polyurea, ketosis, and cardiovascular disorders [2, 3].

Pharmacological treatment of diabetes (hyperglycemia) relies on oral antihyperglycemic agents and insulin but these approaches currently used in clinical practice do not restore normal glycemic levels in most patient. Moreover, continuous use of synthetic antidiabetic drugs has toxicity and causes side effects such as liver problems and diarrhea [2, 3].

Medicinal plants continue to be an important therapeutic aid for humankind. In the last few years there is a growing interest in phytotherapy due to the natural origin and less side effects [4–6].

Nowadays clinical trials are carried out to test the hypoglycemic activity of medicinal herbs. These plants contain biologically active substances so they can be used for therapeutic purposes. Biological action of the medicinal plants is related to active chemical constituents (alkaloids, flavonoids, polyphenols, tannins, triterpenoids, coumarins, and glycosides) usually extending positive effects, because the medicinal properties of plants are due to those chemical constituents [5–7]. The herbal components have similar mechanisms of action as antidiabetic drugs but plants are preferred mainly because of low cost and less side effects.

*Stevia rebaudiana* (SR) is a medicinal herb which has been used in traditional Armenian medicine to decrease blood pressure, glucose, and cholesterol levels, as well as modulate immune function. The first report of commercial cultivation of SR in Paraguay was several decades ago [8–10]. Currently this plant is cultivated on a commercial scale in Armenia using hydroponic method [11]. The latter gives a good reproducible plant material [12]. Indeed, SR containing a variety of secondary metabolites such as glycosides (steviosides), flavonoids, different vitamins (C, A, E, and B), tannins, and others provided a potential source for the treatment of DM [10, 13, 14] but direct effects of these constituents as therapeutic agents should be clearly shown.

The major component of SR is stevioside demonstrating an antihyperglycemic effect in hyperglycemic animals possibly via the reduction of blood glucose levels [15, 16]. Many studies have reported that SR might be a potential choice for DM [17–21]. However, the effects should be clarified, and the specific biochemical mechanisms are not clear.

In the present study we have evaluated some biochemical properties, especially the antihyperglycemic and antihyperlipidemic activity of SR aqueous extract in hyperglycemia induced by immobilization stress in rabbits after 15 days of oral treatment.

## 2. Materials and Methods

### 2.1. Plant Material

SR was grown using hydroponics method [11]. Sprouts of this plant were transplanted in conditions of a classical hydroponics (seating density was 1 plant per cm^2^). As substrate for plant, particles of volcanic slag with diameter of 3–15 mm served; nutrition solution used was, as described [11].

### 2.2. Animals

Hypoglycemic activity of SR extract was carried out on rabbits with the same sex (weighing 1400–1500 g). Initial body weights were recorded one day before the start of commencements of experiments. The animals were kept under standard environmental conditions (temperature 22 ± 2°C in a light/dark cycle of 12 h). The rabbits had free access to food and water during the experimental period. All experiments were performed in accordance with the current ethical norms stated by “International Recommendation on Carrying out of Biomedical Researches with Use of Animals,” and the study plan has been approved by the National Center of Bioethics (Armenia).

### 2.3. Induction of Hyperglycemia in Experimental Rabbits and Blood Sampling

Hyperglycemia was induced immobilizing stress in the rabbits during 15 days (3 h daily) [22, 23]. They were roughly fixed on the board. Group 1 served as hyperglycemic, group 2 was hyperglycemic control (putting immobilization), group 3 in common with immobilization was administrated (got fixed) in single oral doses in 2 mL aqueous extract of SR. Blood glucose levels, lipid profile, and body weight of rabbits were measured at the beginning of the experiment and then on the 1st, 5th, 10th, and 15th days of oral treatment. At the end of experiment the animals were sacrificed, and analysis of liver and muscle glycogen content was carried out.

Blood samples were taken from the aural vein and collected in serum separation tubes (Huma Tube K3E, Germany). Blood clot was removed by centrifugation at 3000*g* for 10 min in a centrifuge at 4°C. The resulting supernatant was designated as a serum.

### 2.4. Study Design

The animals were divided into three groups (*n* = 9) as follows: group 1: nonhyperglycemic, group 2: hyperglycemic control, putting immobilization, and group 3: hyperglycemic experimental, received SR extract (100 mg/kg body weight.). This number of animals was chosen because they showed reliable reproducible results.

### 2.5. Biochemical Analysis

The biochemical analysis was performed to measure the serum level of glucose, total cholesterol (TC), high-density lipoprotein (HDL), low-density lipoprotein (LDL), and triglycerides (TG). All parameters were assayed using enzymatic kit. Serum glucose level (mmol/L) was determined using glucose test kit based on the glucose oxidase method (Dialab Glucose, GOD-PAP, Austria), as described [24]. Total cholesterol and triglycerides were estimated by the method, as developed before [25]. HDL and LDL were measured using the method, as described [26]. The atherogenic index (AI) was determined by the formula, as suggested [26]. Briefly, AI = (TC − HDL)/HDL. Analytical tests were conducted using an UV-Vis spectrophotometer (Genesys 10S, USA).

### 2.6. Histopathological Examination

The liver and muscles of experimental animals were harvested and followed by the histopathological examination; glycogen contents were determined by the method, as described [27].

### 2.7. Data Processing

All values were expressed as ± standard error of the mean. Data processing was done using “Statistica 6.0” software for Windows. The differences between the results of different series were considered valid if Student's criteria (*p*) were <0.05.

## 3. Results

### 3.1. Effect of SR on Fasting Glucose Levels

**Figure 1:**
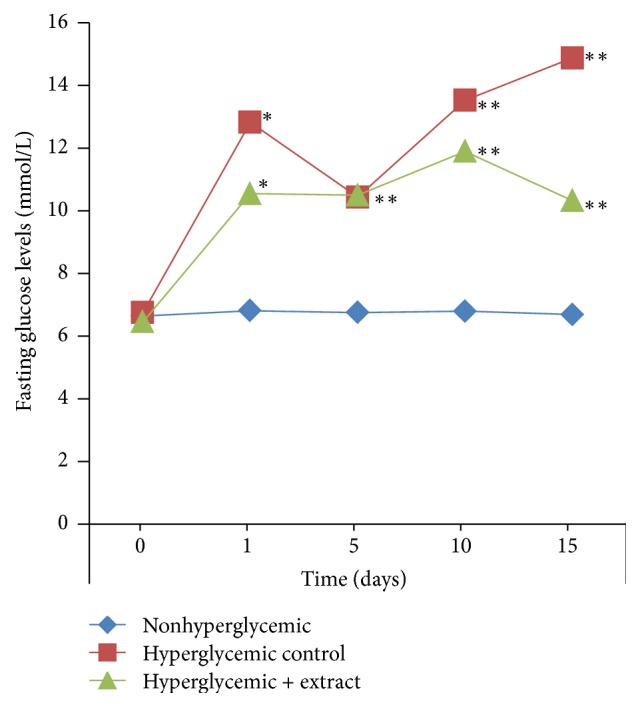
The effects of SR aqueous extract on fasting blood glucose levels in nonhyperglycemic and hyperglycemic rabbits. All data are expressed as mean ± SEM for 3 animals per group. For details see Materials and Methods and the text. ^*∗*^Significantly different levels compared to the nonhyperglycemic group (*p* < 0.05). ^*∗∗*^Significantly different levels compared to the hyperglycemic control group (*p* < 0.05).

The effects of SR aqueous extract on fasting glucose levels in animal blood at different days were determined. The obtained data showed that during the first day of immobilization (3 h) fasting blood glucose levels in the hyperglycemic control (47.0%) and hyperglycemic experimental (+extract) (38.8%) groups were increased, compared to the nonhyperglycemic group (Figure 1).

Therefore, it may be noted that disposable strong stressful pressure provokes hyperglycemia. Fasting glucose level in the hyperglycemic control group significantly increased on the 15th day of immobilization (55.0%) compared to the nonhyperglycemic group. In the group of animals which got the aqueous extract of SR reduction in fasting glucose level was demonstrated at the 15th day reaching the 1st day level (see Figure 1).

### 3.2. Effect of SR on Serum Lipids

Lipid profiles of the experimental animals were investigated (Table 1).

The data showed that the total cholesterol and LDL-cholesterol levels in the hyperglycemic control group were significantly increased (79% and 90%, resp.), compared to the nonhyperglycemic group (see Table 1). The hyperglycemic + extract group demonstrated significantly decreased total cholesterol and LDL-cholesterol levels (66% and 92%, resp.), compared to the hyperglycemic control group. Treatment with SR extract increased HDL-cholesterol level, compared to the hyperglycemic control and nonhyperglycemic rabbits (78%). Although the hyperglycemic control group of animals demonstrated a tendency towards increased LDL-cholesterol level compared to the other groups, AI of hyperglycemic control group significantly increased, compared to the nonhyperglycemic group (94.4%).

### 3.3. Effect of SR on Liver Glycogen Levels

**Figure 2:**
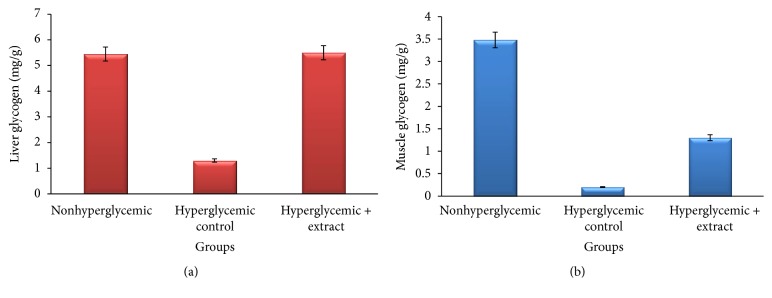
The effects of SR aqueous extracts on liver (a) and muscle (b) glycogen levels. All data are expressed as mean ± SEM for 3 animals per group. For details see Materials and Methods and the text.

The liver and muscle glycogen levels in the various groups are shown in Figure 2. The hyperglycemic control group showed the 4.2-fold reduction in liver glycogen levels, compared to the nonhyperglycemic group (*p* < 0.01). The hyperglycemic + extract group indicated significant increases in the liver glycogen levels (4.2-fold, *p* < 0.01) compared to the hyperglycemic control group (see Figure 2(a)). There were no significant differences in increases in the liver glycogen levels between the treated hyperglycemic group and the nonhyperglycemic group. The hyperglycemic control group showed the strong reduction (19-fold, *p* < 0.01) in muscle glycogen levels, compared to the nonhyperglycemic group (see Figure 2(b)).

The treated hyperglycemic rabbits demonstrated significant increases in the liver glycogen levels compared to the untreated hyperglycemic rabbits. This may suggest that SR extract stimulated insulin secretion from pancreatic *β*-cells, therefore enhancing the impaired capacity of the liver to synthesize glycogen. The treated hyperglycemic rabbits demonstrated the increases in the muscle glycogen levels compared to the hyperglycemic control group (see Figure 2); however the glycogen level muscle can be partly restored. Therefore, it may be suggested that due to immobilization the muscle glycogen was hardly restored.

### 3.4. Body Weight Change

**Figure 3:**
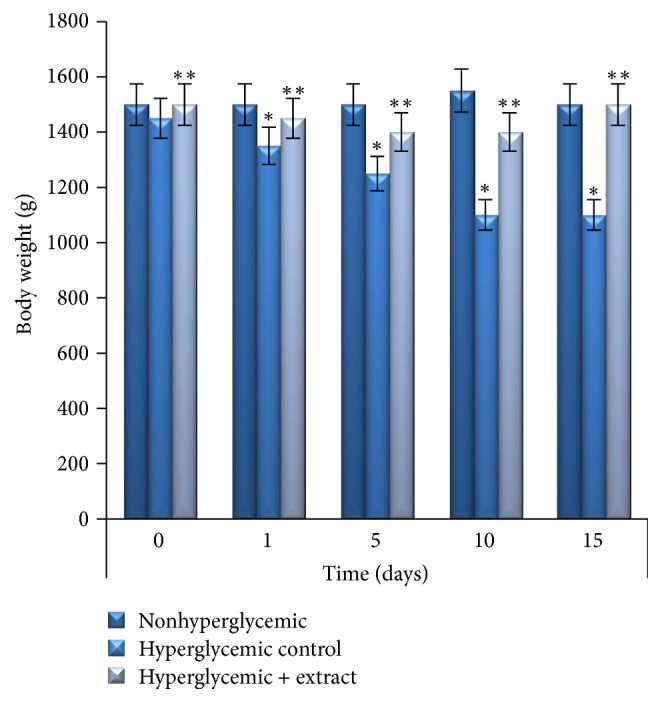
The effects of SR aqueous extract on the body weight of rabbits.  ^*∗*^Significantly different levels compared to the nonhyperglycemic group (*p* < 0.01). ^*∗∗*^Significantly different levels compared to the hyperglycemic control group (*p* < 0.01).

Then, it is known that DM leads to severe body weight loss. During the experimental period body weight change was observed (Figure 3).

There were no significant differences between the pretreated body weights of the various hyperglycemic experimental groups but there was a significant decrease (24%, *p* < 0.01) of the body weights in the hyperglycemic control group, compared to the nonhyperglycemic and extract-treated rabbits (see Figure 3). It should be noted that there were no differences of food consumption between the groups.

## 4. Discussion

Immobilization stress leads to disorder the endocrine, cardiovascular, and immune system. Immobilization is characterized by hyperglycemia, hyperlipidemia, and body weight loss. Disorders of carbohydrate and lipid metabolism lead to the increase of LDL-cholesterol levels and the decrease of HDL-cholesterol levels. For regulation of metabolic disorder phytotherapy is preferred.

The present study investigated the effects of a medicinal plant, namely, SR extract on blood glucose, serum lipids, and glycogen levels in hyperglycemia induced by immobilization stress in rabbits for 15 days. This has demonstrated that 15 days of oral administration of SR extract reduced blood glucose and lipids levels and restored liver glycogen in hyperglycemic rabbits. In the hyperglycemic rabbits compared to the nonhyperglycemic ones it was noted that disposable strong stressful pressure provokes hyperglycemia. SR treatment significantly reduced fasting glucose levels (see Figure 1). The significant reduction of fasting glucose levels by the aqueous extract of SR in the hyperglycemic rabbits may be due to the stimulation of the pancreatic mechanism and probably by increased liver glycogen synthesis and decreased gluconeogenesis [21, 28].

It is known that hyperglycemia is commonly associated with disturbance of lipid metabolism, leading to the increased TC and low-density lipoprotein as well as decreased high-density lipoprotein levels [6, 29–32]. The elevated levels of TC and LDL could be risk factors for cardiovascular disease; conversely, the increased HDL levels, which play a key role in cholesterol transport from the periphery to the liver, reduce the risk of cardiovascular disease [6, 32].

Our results revealed that treatment with SR aqueous extract significantly decreased TC, TG, and LDL levels and increased HDL level in treated hyperglycemic rabbits. It has been reported that most drugs which were used in the treatment of hypercholesterolemia decrease both total and HDL-cholesterol levels [7, 32]. However, SR aqueous extract reduced total cholesterol level and increased HDL-cholesterol level (see Table 1).

The liver is known to be involved in the uptake and metabolism of free fatty acids as well as synthesis of cholesterol, triglycerides, and phospholipids; triglycerides are hydrolyzed by the lipoprotein lipase which is activated by insulin [31]. However, in the diabetic state lipoprotein lipase is not activated due to insulin deficiency which results in increasing hepatic synthesis of triglycerides [32].

It is suggested that insulin stimulates glycogen synthase activity and inhibits glycogenolysis in the liver having a key role for endogenous glucose production [28, 33]. Our study has shown that as the result of immobilization liver and muscle glycogen levels were reduced which could be attributed to a decrease of glycogen synthase activity because of low level of insulin. However, treated animals had increased liver glycogen level, compared to the untreated rabbits (see Figure 2(a)). It may be suggested that SR aqueous extract stimulated insulin secretion from pancreatic *β*-cells.

Then, induction of hyperglycemia is characterized with loss of body weight due to the increased catabolism of protein as a result of insulin deficiency which increased muscle wasting and loss tissue proteins [7, 32]. Treatment with SR aqueous extract improved body weights compared to the hyperglycemic rabbits, which was possibly due to improved glycemic control.

## 5. Conclusions and Significance 

Our investigations revealed that the aqueous extract of SR significantly reduced the levels of some biochemical characteristics in rabbits, especially fasting glucose levels, serum triglycerides, LDL-cholesterol, total cholesterol levels, and increased HDL-cholesterol levels. The extract also increased liver and muscle glycogen content and improved body weights. Therefore, the aqueous extract of SR is suggested to produce antihyperglycemic and antihyperlipidemic activity and to restore liver and muscle glycogen levels (hepatoprotective effects) in hyperglycemia induced by immobilization stress in rabbits. This plant extract might be recommended for treatment of DM; a further study is required.

## Figures and Tables

**Table 1:** The effects of SR aqueous extracts on serum lipids in rabbits.

Characteristics	Experimental groups of animals		
	Nonhyperglycemic	Hyperglycemic control	Hyperglycemic + extract
Total cholesterol (mmol/L)	1.29 ± 0.04	6.21 ± 0.17^*∗*^	2.08 ± 0.06^*∗*^
Triglycerides (mmol/L)	0.98 ± 0.05	1.16 ± 0.07^*∗*^	0.61 ± 0.04^*∗*^
HDL-cholesterol (mmol/L)	0.30 ± 0.06	0.31 ± 0.18^*∗*^	1.46 ± 0.06^*∗*^
LDL-cholesterol (mmol/L)	0.54 ± 0.06	5.83 ± 0.31^*∗*^	0.43 ± 0.05^*∗*^
Atherogenic index	0.29 ± 0.01	5.21 ± 0.31^*∗*^	1.07 ± 0.05^*∗*^

^*∗*^Significantly different from nonhyperglycemic group (*p* < 0.05). All data are expressed as mean ± SEM for 3 animals per group.

## Abbreviations

DM: Diabetes mellitus
SR: * Stevia rebaudiana*
TC: Total cholesterol
LDL: Low-density lipoprotein
HDL: High-density lipoprotein
TG: Triglycerides
AI: Atherogenic index.
